# Fermentative products and bacterial community structure of C_4_ forage silage in response to epiphytic microbiota from C_3_ forages

**DOI:** 10.5713/ab.21.0543

**Published:** 2022-04-29

**Authors:** Siran Wang, Tao Shao, Junfeng Li, Jie Zhao, Zhihao Dong

**Affiliations:** 1 Institute of Ensiling and Processing of Grass, College of Agro-Grassland Science, Nanjing Agricultural University, Nanjing 210095, China

**Keywords:** Bacterial Community, Ensiling, Fermentative Products, Sorghum, Silage

## Abstract

**Objective:**

The observation that temperate C_3_ and tropical C_4_ forage silages easily produce large amounts of ethanol or acetic acid has puzzled researchers for many years. Hence, this study aimed to assess the effects of epiphytic microbiota from C_3_ forages (Italian ryegrass and oat) on fermentative products and bacterial community structure in C_4_ forage (sorghum) silage.

**Methods:**

Through microbiota transplantation and γ-ray irradiation sterilization, the irradiated sorghum was treated: i) sterile distilled water (STSG); ii) epiphytic microbiota from sorghum (SGSG); iii) epiphytic microbiota from Italian ryegrass (SGIR); iv) epiphytic microbiota from oat (SGOT).

**Results:**

After 60 days, all the treated groups had high lactic acid (>63.0 g/kg dry matter [DM]) contents and low pH values (<3.70), acetic acid (<14.0 g/kg DM) and ammonia nitrogen (<80.0 g/kg total nitrogen) contents. Notably, SGIR (59.8 g/kg DM) and SGOT (77.6 g/kg DM) had significantly (p<0.05) higher ethanol concentrations than SGSG (14.2 g/kg DM) on day 60. After 60 days, *Lactobacillus* were predominant genus in three treated groups. Higher proportions of *Chishuiella* (12.9%) and *Chryseobacterium* (7.33%) were first found in silages. The ethanol contents had a positive correlation (p<0.05) with the abundances of *Chishuiella*, *Acinetobacter*, *Stenotrophomonas*, *Chryseobacterium*, and *Sphingobacterium*.

**Conclusion:**

The epiphytic bacteria on raw materials played important roles in influencing the silage fermentation products between temperate C_3_ and tropical C_4_ forages. The quantity and activity of hetero-fermentative *Lactobacillus*, *Chishuiella*, *Acinetobacter*, *Stenotrophomonas*, *Chryseobacterium*, and *Sphingobacterium* may be the key factors for the higher ethanol contents and DM loss in silages.

## INTRODUCTION

Ensiling is considered an excellent preservation method for storing green forage since this method conserves the nutritive components and improves the forage palatability for livestock [1]. In silage production, most of raw plant materials can be easily classified into two categories, namely C_3_ and C_4_ forages. They have evolved in diverse climate patterns, resulting in their different structure, function, and climatic requirements [2]. C_3_ forages, like Italian ryegrass (IR) and oat (OT), have been widely cultivated due to their high palatability when fed as silages. Regarding C_4_ forages, such as maize and sorghum (SG), they could provide higher biomass production and energy as feedstuff. Furthermore, C_3_ forages have a temperate origin, while C_4_ forages have evolved in tropical and arid environments [3].

In well-preserved silages, lactic acid is usually the major fermentation product irrespective of raw materials, however, occasionally there is a big difference in fermentative products between temperate C_3_ and tropical C_4_ forage silages. Li and Nishino [4] found that large amounts of 2,3-butanediol (43.6 g/kg dry matter [DM]) and ethanol (36.2 g/kg DM) existed in wilted IR silage, while acetic acid (21.2 g/kg DM) fermentation occurred in wilted guinea grass (tropical C_4_ forage) silage after 120 days of fermentation. Moreover, Driehuis and van Wikselaar [5] reported that four of twenty-one laboratory silages (mainly *Lolium perenne*, temperate C_3_ forage) were identified as ethanol silages, because their average contents of ethanol reached to 48.1 g/kg DM, while lactic acid contents were just 15.5 g/kg DM. Hence, the problem that temperate C_3_ and tropical C_4_ forage silages easily produce large amounts of ethanol or acetic acid has puzzled researchers for many years. This instability is highly correlated with their anatomical structure, chemical compositions, and microbial properties in raw materials. In plant tissue, the structure of tropical C_4_ forage is more stemmy and coarse, leading to massive air trapped in silo and less dense [6]. In substrate, the water-soluble carbohydrates (WSC) in temperate C_3_ forages mainly include fructose, fructans, sucrose and glucose, whereas tropical C_4_ forages have a large amount of starch [7]. In microbiology, Duniere et al [8] found the epiphytic bacteria on different forages varied greatly, but there is limited information comparing their contributions to fermentative products between temperate C_3_ and tropical C_4_ forages.

Recently, some studies assessed the single contribution of exogenous microbiota to silage fermentation products [9,10]. They used γ-ray irradiation sterilization technology to separate substrate and epiphytic microbes on forages, and selected microbiota transplantation method to explore the individual influence of exogenous epiphytic microbiota on fermentative profiles. However, Nazar et al [9] just assessed the impacts of epiphytic microbiota from different tropical C_4_ forages on silage quality and bacterial community. Wang et al [10] focused on studying the impact of epiphytic microbiota from gramineous grasses on fermentative products of legume forage. Therefore, it is still unclear whether the epiphytic microbiota from temperate C_3_ forages could fit well and reconstitute in tropical C_4_ forage silages. We assumed that transplanting the epiphytic microbiota from temperate C_3_ forages into tropical C_4_ forage silages could reconstitute a bacterial community with similar functions as found in temperate C_3_ forage silages.

Nowadays, SG is becoming an increasingly important C_4_ forage crop in many regions of the world due to its high productivity and ability to utilize water efficiently even under drought conditions [11]. As the main temperate C_3_ forages, IR and OT occupy a large proportion in silage production of temperate areas. Hence, this study aimed to assess the impacts of epiphytic bacteria from OT and IR on fermentation products and bacterial community structure in SG silage. Knowledge regarding the effects of epiphytic microbiota could provide more insights into understanding the microbial factors that cause the differences between temperate C_3_ and tropical C_4_ forage silages.

## MATERIALS AND METHODS

### Inoculum preparation and silage making

Sorghum, IR, and OT were cultivated in the experimental field (Baima Teaching and Research Base) of Nanjing Agricultural University (32°2′N, 118°50′E). This area has a subtropical monsoon climate with an average temperature of 15.7°C, mean annual precipitation of 1,105 mm and average elevation of 24.8 m. Fresh SG was harvested at soft dough stage, while IR and OT were harvested at the heading stage of maturity. Without wilting, each of the three forages was separately chopped (length: 1 to 2 cm) by a chaff cutter (93ZT-300; Xingrong, Guangzhou, China) and made a homogeneous mixing for preparing inoculum and making silage.

The inoculum of SG, IR, and OT were prepared according to the method of Mogodiniyai Kasmaei et al [12] with small modifications. Specifically, 900 mL Ringer solution with added Tween-80 (concentration: 0.5 mL/L) was mixed with 111 g fresh forage. Considering the loss, the nearly 100% epiphytic microbiota on 100 g fresh material could be theoretically represented by the eluted liquid from 111 g fresh material. It was calculated based on two important assumptions in the previous studies [10,12]: i) epiphytic microorganisms were completely eluted from raw material and uniformly distributed in the eluent; ii) after centrifugation, the microbial recovery was 90%. Subsequently, the mixed samples were put in the shaker (rate: 120 rpm; time: 1.5 h) and centrifuged at 12,000×g for 10 min. After centrifugation, the supernatant in tubes was abandoned, and the residues were combined and dissolved in 3 mL Ringer solution. Thus, this 3 mL inoculum collected from 111 g fresh forage represented the whole epiphytic microbiota on 100 g fresh material.

After chopping, fresh SG (100 g) was packed into the vacuum-packed bag (30×32 cm) and sealed with a vacuum sealer. Then, 72 vacuum-packed bags (4 treatments×3 replicates×6 ensiling time = 72) were immediately transported to the irradiation company (Nanjing Xiyue Irradiation Technology Co., Ltd., Wuhu, Anhui, China) by car within 2 h. The samples were sterilized by using a ^60^Co source (dose: 32 kGy; time: 15 min) according to the description of Junges et al [13] To equally evaluate the effects of different epiphytic microbiota on the same substrate, our experiment was conducted based on the equivalent principle that “100 g raw material should be inoculated by the whole epiphytic microbiota from the 100 g fresh material”. Hence, 100 g sterilized SG herein was inoculated by the prepared 3 mL inoculum from SG, IR, and OT, respectively. As the substrate, 100 g irradiated SG was inoculated by the following: i) sterile distilled water (STSG); ii) epiphytic microbiota from sorghum (SGSG); iii) epiphytic microbiota from Italian ryegrass (SGIR); iv) epiphytic microbiota from oat (SGOT). The prepared 3 mL exogenous microbiota or sterile distilled water was added to the irradiated SG. The abovementioned operations were accomplished in the pretreated clean bench, which was irradiated by ultraviolet light for 2 h and cleaned by ethanol to avoid contamination. After inoculation, the vacuum-packed bags were evacuated and sealed. During the inoculating, vacuuming, and sealing, the microbial contamination from the air of the lab were negligible. Finally, the sealed samples were conserved at room temperature (~26°C), and randomly opened after 1, 3, 7, 15, 30, and 60 days of fermentation.

### Fermentation quality analyses

When opening the bags, each sample was mixed thoroughly by hands with sterile gloves in a clean plastic container. The plastic container was irradiated by ultraviolet light for 1 h on the clean bench in advance. Firstly, 25 g sample was blended with 75 mL distilled water and preserved at 4°C for 6 h. Then, we filtered the extracts by one filter paper and two layers of cheesecloth. The filtrates were used for the following analyses. The pH of sample was determined by a glass electrode pH meter (PHSJ-5; LEICI, Shanghai, China). The buffering capacity of fresh forage were determined according to the method of Playne and McDonald [14]. Then, the filtrate was stored at −20°C for analyzing ethanol, organic acids, and ammonia nitrogen (NH_3_-N) contents. The organic acid and ethanol contents were determined with the Agilent HPLC 1260 (Agilent Technologies, Inc., Santa Clara, CA, USA; column: Carbomix H-NP5, Sepax Technologies, Inc., Santa Clara, USA; detector: refractive index detector, Agilent Technologies, Inc., USA; eluent: 2.5 mmol/L H_2_SO_4_, 0.5 mL/min; temperature: 55°C). The NH_3_-N contents were determined according to the description of Broderick and Kang [15].

Secondly, a part of fresh forage or silage was analyzed immediately for the DM content in a forced-draft oven to a constant weight drying at 60°C for at least 48 h, and then ground to pass a 1 mm screen in a laboratory knife mill (FW100; Taisite Instrument Co., Ltd., Tianjin, China) for later analysis. The milled sample was used for total nitrogen (TN), WSC, and fiber analysis. The TN concentrations were determined according to Kjeldahl method [16]. The WSC contents were measured through the method of anthrone colorimetry [17]. The acid detergent fiber (ADF) and neutral detergent fiber (NDF) contents were determined by the method of Van Soest et al [18].

Thirdly, for enumeration of the microorganisms, 10 g pre-ensiled sample or silage was shaken well with 90 mL of sterilized saline solution (0.85% NaCl) at 120 rpm for 1 h. Then 1 mL solution was used for 10-fold serial dilution for microorganism counting, and then the remaining solution was filtered through 4 layers of medical gauze and stored in the −80°C refrigerator for DNA extraction. The colonies of lactic acid bacteria (LAB) were counted on MRS agar medium after incubation in an anaerobic incubator (N_2_:H_2_:CO_2_ = 85:5:10, YQX-II; CIMO Medical Instrument Manufacturing Co., Ltd., Shanghai, China) at 37°C for 3 days. Aerobic bacteria were cultured and counted on nutrient agar medium (Nissuiseiyaku Ltd., Tokyo, Japan). Yeasts were counted on potato dextrose agar (Nissui-seiyaku Ltd., Japan) and acidified with sterilized tartaric acid solution to pH 3.5. These agar plates were incubated at 37°C for 3 days. Enterobacteriaceae was counted on the Violet Red Bile Glucose Agar medium after 24 h of incubation at 37°C under aerobic conditions.

### Bacterial community analysis

The bacterial community varies greatly at the initial stage of fermentation, and the final state of silage is important for researchers to evaluate the fermentation quality. Hence, the raw materials (SGFM, IRFM, OTFM), and silage samples on day 3 (SGSG-3, SGIR-3, SGOT-3) and day 60 (SGSG-60, SGIR-60, SGOT-60) were selected to investigate their bacterial diversity through high throughput sequencing. The details in determining bacterial diversity of these samples were according to the description of Wang et al [19]. In brief, the preserved liquid for extracting DNA was centrifuged (rate: 12,000×g; time: 10 min), and pellet was collected and used to extract DNA by kit (MP Biomedicals, Santa Ana, CA, USA) based on the description of manufacture’s protocols. The 338F and 806R were selected as primers to amplify the V3-V4 regions of bacterial 16S ribosomal RNA gene. DNA samples were paired-end sequenced on the platform of Illumina MiSeq PE300.

The FLASH software was applied to check the raw reads, and the quality sequences (scores >80) were saved based on the QIIME quality control process. The operational taxonomic units (OTUs) with 97% similarity were clustered by UPARSE pipeline software. Then we used UCHIME software to identify and remove the chimeric sequences. The alpha-diversities including rarefaction curves, Shannon curves, Shannon, Chao1, Sobs, Simpson, Coverage and Ace indexes were performed using Mothur software. The bacterial community compositions were determined at genus and phylum levels through the Silva 138 database (confidence: >70%). The spearman correlation heatmap and hierarchical cluster analysis were graphically displayed by R software (Version 4.0.5).

### Statistical analysis

The Statistical Packages for the Social Sciences (SPSS, version19.0, SPSS Inc., Chicago, IL, USA) was used to examine the differences among treatments. The comparison between fresh and sterile SG was conducted by one-way analysis of variance (ANOVA). Data on fermentative characteristics and microbial populations was analyzed via two-way ANOVA. The microbial populations were acquired as colony-forming units (cfu) based on fresh weight (FW). The method of Tukey’s multiple comparison was applied to analyze the statistical difference. Differences were regarded as significant at p<0.05.

## RESULTS AND DISCUSSION

Ensiling is an anaerobic fermentation process in which microbes are involved and play important roles in determining the silage quality by interacting with chemical compositions contained in the raw materials. On the one hand, the nutrients in the raw material could be effectively conserved via ensiling; on the other hand, the microbes involved in anaerobic fermentation also generate various metabolites that could alter the nutritional structure. To investigate the contribution of various microbiota to the fermentative products in silage, the dynamic chemical compositions and bacterial communities during SG ensiling were evaluated.

### Chemical components and microbial populations of sterile and fresh sorghum

The WSC concentration of raw material is one of conclusive factors for silage making. The WSC is the fermentable substrate during ensiling, which is chiefly used by LAB to generate organic acids, thus reducing the silage pH value. To obtain the quality silage, the optimal WSC content of raw material should be higher than 50 g/kg DM [20]. As shown in Table 1, the WSC content (173 g/kg DM) of fresh SG satisfied the requirement. This indicated that SG is suitable for assessing the fermentative products and bacterial community dynamics in response to exogenous microbiota because sufficient fermentation substrate can be provided for microorganisms during ensiling.

The chemical compositions between sterile and fresh SG were similar (p>0.05). It was suggested that the sterilized condition used would not significantly change the chemical compositions of raw material. This is the prerequisite for evaluating the single contribution of exogenous microbiota to fermentation quality on the same raw material. Similarly, Wang et al [19] reported that the γ-ray irradiation condition (32 kGy for 4 h) would not obviously alter the chemical compositions and enzyme properties of substrate, and γ-ray irradiation method was more efficient than heating and chemical additives. Furthermore, the epiphytic microbial populations on sterile SG were not detected. This indicated that the method of γ-ray irradiation sterilization could guarantee that the epiphytic microbes of raw material are inactivated after irradiation.

### The alpha diversities of fresh materials and silages

**Figure 1:**
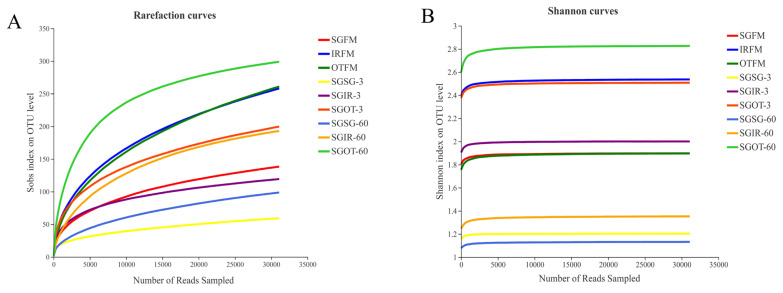
Rarefaction curves (A) and Shannon curves (B) of the bacterial community in fresh materials and sorghum silages. SGFM, fresh material of sorghum; IRFM, fresh material of Italian ryegrass; OTFM, fresh material of oat; SGSG, sterile sorghum inoculated by epiphytic bacteria from sorghum; SGIR, sterile sorghum inoculated by epiphytic bacteria from Italian ryegrass; SGOT, sterile sorghum inoculated by epiphytic bacteria from oat. 3, 3 days of ensiling; 60, 60 days of ensiling.

In microbial ecology, rarefaction curves are often applied to evaluate the richness of microbial community according to the sequencing results. As found in Figure 1(A), with the number of reads sampled increasing, the rarefaction curves in all samples exhibited an upward trend during the initial stage of detection, and then slowly increased at the end. This indicated that the amount of sequencing data produced was reasonable and can fully reflect the diversity of bacterial community in each sample. More sequencing data (>30,000) would only generate a few species (OTU) that would not influence the overall evaluation of bacterial community structure in samples. As described in Figure 1(B), the Shannon curves reached to stable levels at the early stage of detection. This proved that the sequencing data obtained could provide most of the information about the bacterial communities in samples.

Alpha diversity parameters, such as Shannon, Sobs, Simpson, Chao1, Coverage, and Ace indices could reflect the richness, diversity, and coverage of the bacterial community in samples. As described in Table 2, fresh materials had higher alpha diversities than silage samples, especially in indices of Sobs, Ace and Chao1. It was probably because the acidic and anaerobic environment in silage samples was rapidly formed during ensiling, leading to a remarkable decrease in the bacterial diversity. This also indicated that the acidic and anaerobic environmental stresses had a great impact on the proliferation and growth of microorganisms in silages. SGSG group had lower indices of Sobs, Shannon and Chao1 than SGIR and SGOT groups on day 3. According to the results in bacterial compositions on genus level, it may be because *Weissella* and *Lactococcus* in SGSG-3 accounted for a large proportion in the bacterial community, resulting in a relatively homogenous community of bacteria. This agreed with the results of Du et al [21], who found that the lowest alpha diversity was observed in the quality silage due to the large proportions of beneficial microorganisms. Notably, SGOT had higher indices of Sobs, Shannon, Ace and Chao1, and lower Simpson indices than SGSG and SGIR on day 60. This indicated that the bacterial community structure in SGOT-60 was more diverse and complicated. The high Coverage values (>99.67%) for all samples suggested that the sampling depth had adequately captured most of the bacterial communities and was sufficient for reliable analysis of the bacterial community.

### The bacterial community compositions in the fresh materials and silages

Among a variety of factors that could influence the ensiling process, the predominant bacterial species usually determine the quality of silage. Hence, analyzing the dynamic changes of bacterial compositions during fermentation can contribute to understanding the ensiling process and improving the silage quality.

**Figure 2:**
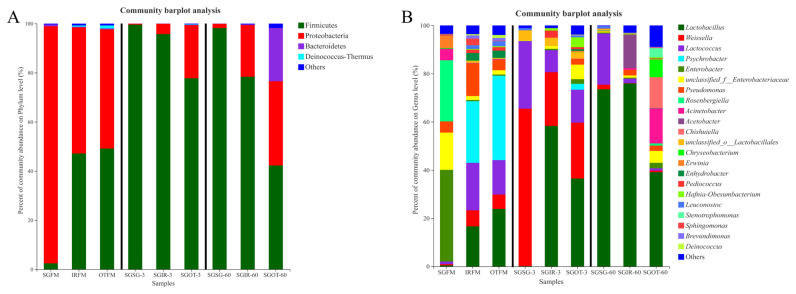
Phylum (A) and genus (B) level compositions of the bacterial community in fresh materials and sorghum silages. SGFM, fresh material of sorghum; IRFM, fresh material of Italian ryegrass; OTFM, fresh material of oat; SGSG, sterile sorghum inoculated by epiphytic bacteria from sorghum; SGIR, sterile sorghum inoculated by epiphytic bacteria from Italian ryegrass; SGOT, sterile sorghum inoculated by epiphytic bacteria from oat. 3, 3 days of ensiling; 60, 60 days of ensiling.

As shown in Figure 2(A), Proteobacteria (51.4%; 48.5%) and Firmicutes (47.1%; 49.2%) were both the predominant phyla in IRFM and OTFM. Proteobacteria are critical in degrading organic matter, and accelerating nitrogen and carbon cycles [22]. Regarding Firmicutes, their acid hydrolytic function plays a vital role in various anaerobic environments, and multifarious enzymes could be produced by Firmicutes, such as proteases, cellulases, and other extracellular enzymes [23]. Unlike IRFM and OTFM, Proteobacteria (>96.4%) was the only dominant phylum in SGFM. This difference might be attributed to the chemical compositions of different fresh materials and environmental factors [19]. After ensiling, the relative abundances of Firmicutes in treated groups increased in varying degrees. It was probably because the anaerobic and acidic environment during the fermentation was conducive to the growth and proliferation of Firmicutes [24].

As found in Figure 2(B), prior to ensiling, higher proportions (38.0%) of *Enterobacter* were observed in SGFM, a finding similar to Wang et al [19], who reported that undesirable molds, yeasts and Enterobacteriaceae are the most abundant epiphytic microbes. The most dominate genus in IRFM (25.6%) and OTFM (35.0%) was *Psychrobacter*, which may be linked with their suitability for growing in humid and cold environment [19].

After ensiling, the proportion of *Weissella* in SGSG (65.4%) was much higher than that in SGIR (22.3%) and SGOT (23.2%) on day 3. *Weissella* are reported as the initial colonizer microorganisms during the ensiling and are obligate hetero-fermentative bacteria that mainly metabolize WSC to acetate and lactate, and acidic condition is adverse to their growth [25]. Thus, the significantly (p<0.05) lower lactic acid contents and higher pH values in SGSG than SGIR and SGOT on day 3 may be attributed to the higher proportions of hetero-fermentative *Weissella* in SGSG-3, because hetero-fermentative LAB had lower efficiency at producing lactic acid [26].

SGIR (58.3%) and SGOT (36.5%) had much higher proportions of *Lactobacillus* than SGSG (0.12%) on day 3. *Lactobacillus* can utilize one molecule of glucose to produce two molecules of lactic acid. Some aerobic microorganisms and plant cells consume oxygen during the initial stage of fermentation, then species of *Lactobacillus* rapidly grow and proliferate, whilst generating a large amount of lactic acid to reduce pH values of silage. At last, pathogenic microorganisms (e.g. *Clostridium*) are restricted [27]. Thus, the massive production of lactic acid in SGIR and SGOT on day 3 may be highly linked with their higher proportions of *Lactobacillus*. Notably, after 60 days of fermentation, the relative abundances of *Lactobacillus* in SGSG and SGIR were 73.5% and 76.0% respectively, while the relative abundance of *Lactobacillus* in SGOT was just 39.1%. Based on the fact that SGIR (59.8 g/kg DM) and SGOT (77.6 g/kg DM) had significantly (p<0.05) higher ethanol contents than SGSG (14.2 g/kg DM) on day 60, it was speculated that the *Lactobacillus* in SGIR-60 may be mainly responsible for the massive production of ethanol through the hetero-fermentative pathway, while the higher ethanol contents in SGOT-60 were more related to the metabolism of undesirable microorganisms rather than *Lactobacillus*.

It is well documented that an acidic environment is adverse to cocci-LAB strains, while a large proportion of *Lactococcus* (21.3%) was found in SGSG after 60 days of ensiling. It was suggested that some *Lactococcus* may promote the accumulation of lactic acid even at the end of ensiling. This agreed with the findings of Nishino et al [28], who reported that some cocci LAB strains could exist over the fermentation process in some silages. Furthermore, a high proportion of *Acetobacter* (13.9%) was observed in SGIR on day 60. Liu et al [29] reported that *Acetobacter* might result in the aerobic spoilage of silages after exposure to air. Moreover, genus *Acinetobacter* accounted for a large proportion (14.4%) in SGOT on day 60. Species of *Acinetobacter* could proliferate quickly in the acidic environment and cause aerobic spoilage in silage [20]. The existence of *Acetobacter* and *Acinetobacter* were both adverse to the aerobic stability in silages, more attention should be thus paid on their populations during the silage production.

It is noteworthy that 12.9% of *Chishuiella* and 7.33% of *Chryseobacterium* were observed in SGOT on day 60. Zhang et al [30] found that cells of *Chishuiella* are Gram-reaction-negative, strictly aerobic, rod shaped, and non-gliding, and the genus *Chishuiella* is a member of the family Flavobacteriaceae in the phylum Bacteroidetes. Hugo et al [31] reported that the genus *Chryseobacterium* is classified within the family Flavobacteriaceae, and almost all species are strictly aerobic. Several species of *Chryseobacterium* occur in food or dairy products; they may be involved in spoilage. More importantly, to the best of our knowledge, this is the first time to report the existence of *Chishuiella* and *Chryseobacterium* in silages. Hence, more research about their roles during ensiling needs to be conducted.

### Fermentation characteristics and their correlation with bacterial community compositions

As described in Table 3 and 4, STSG group remained in an unfermented state and had similar (p>0.05) chemical compositions with fresh SG during the ensiling period. This indicated that γ-ray irradiation with optimal dose could successfully separate the microbial and chemical compositions of herbage. The pH value of silage is critical for assessing the silage quality, and lactic acid is desirable during ensiling. During the initial stages of fermentation, lactic acid contents accumulated rapidly, and pH values declined dramatically in three treated groups. It was because forages were chopped into small pieces to ensure the rapid release of plant juice, which promoted the growth, proliferation, and metabolism of LAB at the early stage. SGSG had significantly (p<0.05) lower lactic acid contents and higher pH values than SGIR and SGOT on day 3. It could be ascribed to the higher abundance of hetero-fermentative *Weissella* existed in SGSG-3.

During the entire ensiling process, the contents of acetic acid in three treated groups gradually increased. McDonald et al [1]. explained that the acetic acid primarily come from the metabolites of enterobacteria, *Propionibacterium* and hetero-fermentative LAB on WSC during the ensiling. Trace and acceptable amounts of butyric acids (<2 g/kg DM) in all fermented groups may be owing to the quick decline in pH values at the initial stage of fermentation, inhibiting the growth, proliferation and activity of clostridia and other undesirable microbes.

Greater losses of DM and energy in silages can be resulted from the high production of ethanol. Kung et al [32] found that over 30 to 40 g/kg DM of ethanol content may be linked with the action of yeasts. In the current study, the ethanol contents in SGIR and SGOT on day 60 were higher than 50.0 g/kg DM, suggesting that the ethanol contents in these two groups were mainly generated by acid-resistant yeasts and other microorganisms like hetero-fermentative LAB. Borreani et al [26] reported that hetero-fermentative LAB strains could generate carbon dioxide, lactic acid and some by-products like ethanol and acetic acid. This indirectly proved that the majority of *Lactobacillus* in SGIR-60 belonged to hetero-fermentative LAB strains.

As shown in Table 4, the DM contents in STSG group remained stable compared with the fresh SG. This indicated that appropriate γ-ray irradiation could successfully inactivate the microorganisms in forages, thus preventing the fermentation process. The DM contents of all fermented groups were dramatically (p<0.05) reduced after ensiling, which is indicative of the microbial breakdown of nutrients into carbon dioxide and water. Compared to SGOT and SGIR on day 60, the notably (p<0.05) higher DM contents in SGSG could be ascribed to the less ethanol contents in SGSG-60, thus preserving more nutrients in silages. Similarly, Pahlow et al [33] reported that greater DM recovery in well-preserved silage could be partially explained by the lower ethanol production indicating less inefficient secondary fermentation by yeasts and hetero-fermentative bacteria.

Ammonia nitrogen in silage reflects the level of protein degradation that can reduce the nutritional value of forage. Herein, the generation of NH_3_-N in STSG was primarily linked with the action of plant enzyme. All the fermented silages had lower NH_3_-N concentrations; less than 100 g/kg TN is considered by McDonald et al [34] to be acceptable for good fermentation in silages. This was mainly attributed to the quick decline of pH values during the initial stage of ensiling, suppressing the enzymatic activity in plant and microorganisms.

Beneficial microorganisms could improve the fermentation quality by producing a variety of desirable metabolites. In the current study, SGSG had significantly (p<0.05) lower LAB counts than SGIR and SGOT on day 3. It may partially explain the result that SGSG had less lactic acid contents and lower abundance of *Lactobacillus* than SGOT and SGIR on day 3.

**Figure 3:**
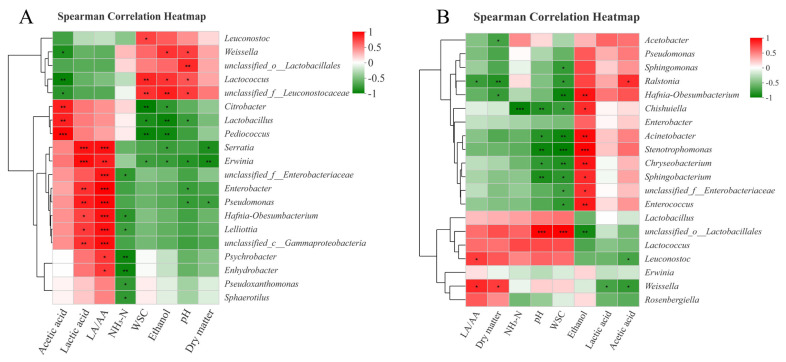
Spearman correlation heatmap between fermentation parameters and bacterial community compositions in sorghum silages on day 3 (A) and day 60 (B). NH_3_-N, ammonia nitrogen; LA/AA, ratio of lactic acid to acetic acid; WSC, water soluble carbohydrate.

The relationships between the bacterial community and fermentative products in SG silages on day 3 and 60 are described in Figure 3. The negative correlation (p<0.05) between abundance of *Lactobacillus* and pH values, and positive correlation (p<0.05) between abundance of *Weissella* and pH values were found on day 3 (Figure 3(A)). This indicated that species of *Lactobacillus* played more important roles than *Weissella* in promoting homo-lactic acid fermentation and reducing pH values during the early stage of ensiling. On day 60 (Figure 3(B)), the ethanol contents had a positive correlation (p<0.05) with the abundances of *Chishuiella*, *Acinetobacter*, *Stenotrophomonas*, *Chryseobacterium*, and *Sphingobacterium*. It indicated that these undesirable microorganisms may indirectly accelerate the production of ethanol during ensiling. For instance, Shankar et al [35] found that the consortium of crude lignocellulolytic enzymes produced by *Sphingobacterium* sp. ksn could enhance the production of ethanol.

## CONCLUSION

The epiphytic bacteria on raw materials played important roles in influencing the silage fermentation products between temperate C_3_ and tropical C_4_ forages. The quantity and activity of hetero-fermentative *Lactobacillus*, *Chishuiella*, *Acinetobacter*, *Stenotrophomonas*, *Chryseobacterium*, and *Sphingobacterium* may be the key factors for higher ethanol contents and DM loss in silages. In practice, to prevent the massive ethanol production in C_4_ silages, more attention should be paid on inhibiting or reducing the activity and quantity of abovementioned undesirable microorganisms.

## Figures and Tables

**Table 1:** Chemical and microbial compositions of fresh and sterile sorghum before ensiling

Items	Fresh sorghum	Sterile sorghum	p-value
Dry matter (g/kg FW)	299	294	0.627
Water soluble carbohydrates (g/kg DM)	173	176	0.583
Buffering capacity (mEq/kg DM)	75.9	73.7	0.332
Neutral detergent fiber (g/kg DM)	570	567	0.923
Acid detergent fiber (g/kg DM)	272	269	0.790
Crude protein (g/kg DM)	72.6	72.0	0.295
Lactic acid bacteria (log_10_ cfu/g FW)	5.72	ND	-
Aerobic bacteria (log_10_ cfu/g FW)	5.36	ND	-
Yeasts (log_10_ cfu/g FW)	4.21	ND	-
Enterobacteriaceae (log_10_ cfu/g FW)	4.89	ND	-

DM, dry matter; FW, fresh weight; mEq, milligram equivalent; cfu, colony-forming units; ND, not detected.

**Table 2:** Richness and diversity indices of bacterial communities in fresh materials and sorghum silages on day 3 and 60

Samples	Sequence number	Sobs	Shannon	Simpson	Ace	Chao1	Coverage
SGFM	46,443	138	1.897	0.250	259	192	0.9985
IRFM	53,538	258	2.536	0.151	324	324	0.9970
OTFM	49,809	351	2.563	0.139	636	503	0.9967
SGSG-3	52,526	59	1.203	0.456	186	125	0.9992
SGIR-3	72,176	119	2.234	0.360	181	166	0.9989
SGOT-3	69,641	223	2.508	0.206	355	341	0.9979
SGSG-60	61,625	99	1.132	0.482	220	177	0.9987
SGIR-60	66,488	246	1.686	0.334	294	283	0.9983
SGOT-60	62,103	299	2.826	0.139	337	335	0.9985

SGFM, fresh material of sorghum; IRFM, fresh material of Italian ryegrass; OTFM, fresh material of oat; SGSG, sterile sorghum inoculated by epiphytic microbiota from sorghum; SGIR, sterile sorghum inoculated by epiphytic microbiota from Italian ryegrass; SGOT, sterile sorghum inoculated by epiphytic microbiota from oat; 3, 3 days of ensiling; 60, 60 days of ensiling.

**Table 3:** Effect of inoculating exogenous microbiota on pH value, organic acid, and ethanol concentrations in sorghum silage

Items	Treatments^1)^	Ensiling days (d)						SEM	p value^2)^		
	
		1	3	7	15	30	60		T	D	T×D
pH value	STSG	5.15^a^	5.17^a^	5.18^a^	5.17^a^	5.21^a^	5.19^a^	0.024	<0.001	<0.001	<0.001
	SGSG	5.16^Aa^	4.44^Bb^	4.32^Bb^	3.91^Cb^	3.63^Db^	3.64^Db^				
	SGIR	5.19^Aa^	3.98^Bc^	3.79^Cd^	3.70^Dc^	3.65^DEb^	3.62^Eb^				
	SGOT	4.19^Ab^	3.96^Bc^	3.93^Bc^	3.74^Cc^	3.64^Db^	3.48^Ec^				
Lactic acid (g/kg DM)	STSG	0.59^b^	0.61^c^	0.60^d^	0.64^c^	0.66^c^	0.69^c^	1.175	<0.001	<0.001	<0.001
	SGSG	0.61^Eb^	13.4^Db^	17.9^Dc^	50.6^Cb^	56.5^Bb^	63.8^Ab^				
	SGIR	1.52^Fb^	34.7^Ea^	52.4^Da^	59.9^Ca^	64.7^Ba^	69.9^Aa^				
	SGOT	14.7^Ea^	35.3^Da^	47.2^Cb^	60.9^Ba^	67.4^Aa^	65.6^ABb^				
Acetic acid (g/kg DM)	STSG	0.13^Bd^	0.26^Bc^	0.26^Bc^	0.23^Bd^	0.57^Ad^	0.75^Ad^	0.524	<0.001	<0.001	<0.001
	SGSG	3.50^Ca^	3.33^Cb^	4.56^Bb^	6.28^Ab^	6.59^Ac^	6.56^Ac^				
	SGIR	0.84^Ec^	5.55^Da^	8.53^Ca^	9.20^Ca^	10.6^Ba^	13.6^Aa^				
	SGOT	2.25^Eb^	3.82^Db^	4.92^Cb^	5.75^Bc^	9.20^Ab^	9.39^Ab^				
Lactic acid/acetic acid	STSG	4.81^Ab^	2.35^Bd^	2.34^Bd^	2.82^Bd^	1.17^Cd^	0.93^Cd^	0.151	<0.001	<0.001	<0.001
	SGSG	0.17^Dc^	4.03^Cc^	3.95^Cc^	8.06^Bb^	8.57^Ba^	9.72^Aa^				
	SGIR	1.78^Cc^	6.28^Ab^	6.15^ABb^	6.52^Ac^	6.10^ABc^	5.13^Bc^				
	SGOT	6.56^Ba^	9.24^Aa^	9.58^Aa^	10.6^Aa^	7.32^Bb^	6.98^Bb^				
Butyric acid (g/kg DM)	STSG	0.81^b^	0.86^c^	0.86^c^	0.81^c^	0.80^d^	0.81^c^	0.235	<0.001	<0.001	<0.001
	SGSG	1.24^Da^	1.74^Ca^	1.81^Ba^	1.87^ABa^	1.91^ABa^	1.94^Aa^				
	SGIR	0.46^Dc^	0.55^Dd^	0.60^Dd^	0.84^Cc^	1.43^Bc^	1.54^Ab^				
	SGOT	0.43^Cc^	1.04^Bb^	1.13^Bb^	1.42^Ab^	1.54^Ab^	1.63^Ab^				
Ethanol (g/kg DM)	STSG	4.17^a^	4.13^b^	4.23^c^	4.28^d^	4.20^d^	4.18^d^	0.281	<0.001	<0.001	<0.001
	SGSG	2.26^Fc^	6.97^Ea^	7.65^Db^	9.74^Cc^	13.2^Bc^	14.2^Ac^				
	SGIR	2.78^Db^	3.98^Db^	12.8^Ca^	22.1^Ba^	57.5^Ab^	59.8^Ab^				
	SGOT	2.51^Ebc^	4.25^Eb^	7.64^Db^	14.2^Cb^	67.1^Ba^	77.6^Aa^				

d, day; SEM, standard error of means; DM, dry matter.

1) STSG, sterile sorghum; SGSG, sterile sorghum inoculated by epiphytic microbiota from sorghum; SGIR, sterile sorghum inoculated by epiphytic microbiota from Italian ryegrass; SGOT, sterile sorghum inoculated by epiphytic microbiota from oat.

2) T, microbiota; D, ensiling days; T×D, the interaction between microbiota and ensiling days.

a–d Means with different letters in the column differ (p<0.05).

A–F Means with different letters in the same row differ (p<0.05).

**Table 4:** Effect of inoculating exogenous microbiota on chemical and microbial compositions in sorghum silage

Items	Treatments^1)^	Ensiling days (d)						SEM	p-value^2)^		
	
		1	3	7	15	30	60		T	D	T×D
Dry matter (g/kg DM)	STSG	294	296	294^a^	296^a^	295^a^	296^a^	1.302	<0.001	<0.001	<0.001
	SGSG	295^A^	294^A^	293^Aa^	288^Ab^	276^Bb^	276^Bb^				
	SGIR	296^A^	290^B^	281^Cb^	275^Dc^	274^DEb^	269^Ec^				
	SGOT	294^A^	290^AB^	288^ABa^	285^Bb^	274^Cb^	262^Dd^				
Water soluble carbohydrates (g/kg DM)	STSG	175^a^	175^a^	175^a^	176^a^	175^a^	175^a^	1.209	<0.001	<0.001	<0.001
	SGSG	173^Aa^	168^Ab^	129^Bb^	122^Cb^	106^Db^	95.8^Eb^				
	SGIR	175^Aa^	132^Bd^	112^Cc^	103^Dc^	102^Db^	92.9^Eb^				
	SGOT	164^Ab^	138^Bc^	126^Cb^	92.7^Dd^	58.2^Ec^	47.2^Ec^				
Ammonia nitrogen (g/kg TN)	STSG	14.6^Ec^	19.4^Ec^	33.9^Db^	47.2^Cc^	57.6^Bc^	64.0^Ac^	1.230	<0.001	<0.001	<0.001
	SGSG	26.6^Fab^	37.4^Ea^	47.9^Da^	58.5^Cb^	66.2^Bb^	79.0^Aa^				
	SGIR	27.4^Ea^	38.3^Da^	44.8^Ca^	71.2B^a^	75.9^ABa^	79.4^Aa^				
	SGOT	21.7^Eb^	27.6^Eb^	32.4^Db^	42.8^Cd^	56.9^Bc^	72.1^Ab^				
Lactic acid bacteria (log_10_/cfu g FW)	STSG	ND	ND	ND	ND	ND	ND	0.122	<0.001	<0.001	<0.001
	SGSG	5.35^Eb^	7.68^Cc^	9.57^A^	9.38^ABc^	8.70^Bb^	6.42^Dab^				
	SGIR	6.35^Ea^	8.29^Cb^	9.15^B^	10.7^Aa^	7.63^Dc^	5.84^Fb^				
	SGOT	5.46^Db^	9.30^Ba^	9.57^B^	10.2^Ab^	9.83^ABa^	6.95^Ca^				
Enterobacteriaceae (log_10_ cfu/g FW)	STSG	ND	ND	ND	ND	ND	ND	0.115	<0.001	<0.001	<0.001
	SGSG	8.23^Ab^	6.57^Bb^	5.30^C^	4.22^Dc^	4.14^Db^	4.26^D^				
	SGIR	9.11^Aa^	7.31^Ba^	5.11^C^	5.24^Ca^	4.16^Db^	4.24^D^				
	SGOT	8.17^Ab^	6.43^Bb^	5.55^C^	4.75^Db^	4.42^DEa^	4.19^E^				

SEM, standard error of means; DM, dry matter; TN, total nitrogen; cfu, colony-forming units; FW, fresh weight; ND, not detected.

1) STSG, sterile sorghum; SGSG, sterile sorghum inoculated by epiphytic microbiota from sorghum; SGIR, sterile sorghum inoculated by epiphytic microbiota from Italian ryegrass; SGOT, sterile sorghum inoculated by epiphytic microbiota from oat.

2) T, microbiota; D, ensiling days; T×D, the interaction between microbiota and ensiling days.

a–d Means with different letters in the column differ (p<0.05).

A–F Means with different letters in the same row differ (p<0.05).
